# Understanding exposomes and its relation with cancer risk in Malaysia based on epidemiological evidence: a narrative review

**DOI:** 10.1186/s41021-024-00300-0

**Published:** 2024-02-08

**Authors:** Razinah Sharif, Theng Choon Ooi

**Affiliations:** Centre of Healthy Ageing and Wellness, Faculty of Health Sciences, Universiti Kebangsaan Malaysia, Bangi, Malaysia

**Keywords:** Exposomes, cancer risk, Malaysia, Dietary carcinogen, Environmental contaminants, Occupational hazards

## Abstract

The prevalence of cancer is increasing globally, and Malaysia is no exception. The exposome represents a paradigm shift in cancer research, emphasizing the importance of a holistic approach that considers the cumulative effect of diverse exposures encountered throughout life. The exposures include dietary factors, air and water pollutants, occupational hazards, lifestyle choices, infectious agents and social determinants of health. The exposome concept acknowledges that each individual’s cancer risk is shaped by not only their genetic makeup but also their unique life experiences and environmental interactions. This comprehensive review was conducted by systematically searching scientific databases such as PubMed, Scopus and Google Scholar, by using the keywords “exposomes (environmental exposures AND/OR physical exposures AND/OR chemical exposures) AND cancer risk AND Malaysia”, for relevant articles published between 2010 and 2023. Articles addressing the relationship between exposomes and cancer risk in the Malaysian population were critically evaluated and summarized. This review aims to provide an update on the epidemiological evidence linking exposomes with cancer risk in Malaysia. This review will provide an update for current findings and research in Malaysia related to identified exposomes-omics interaction and gap in research area related to the subject matter. Understanding the interplay between complex exposomes and carcinogenesis holds the potential to unveil novel preventive strategies that may be beneficial for public health.

## Background

### Understanding exposomes and its relation with carcinogenesis

Cancer is known for its multifactorial etiology and a complex disease continues to be a leading cause of death worldwide. While genetic factors have long been recognized as important contributors to cancer risk, emerging research has shed light on the significant influence of environmental exposures. One field in epidemiology that greatly relies on improved measurements is exposure assessment, and this need has been emphasized through the concept of the exposome. Moreover, the ability to observe genetic and epigenetic changes in individuals exposed to potential risk factors offers an opportunity to understand the underlying mechanisms of cancer development, leading to earlier detection and more precise categorization of the disease at a molecular level.

**Fig. 1:**
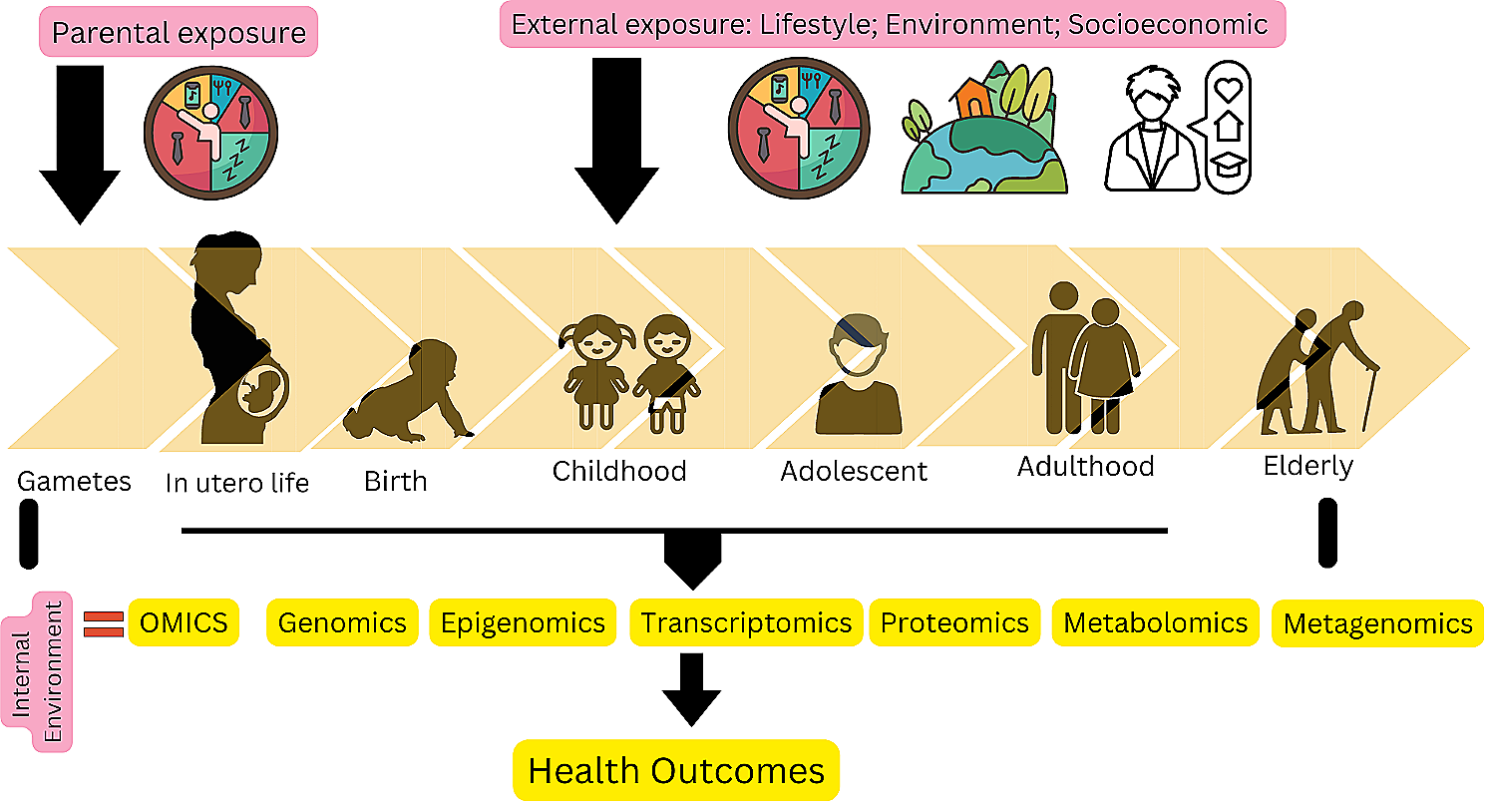
Exposomic approach and carcinogenesis

The exposome signifies a comprehensive assessment of all lifelong environmental influences (including biological, chemical, physical, and psychosocial factors) and the corresponding biological reactions within an individual, in addition to the pre-existing genetic factors. The discussion about exposomes normally will involve 3 different aspects which include internal exposomes (metabolism, endogenous circulating hormones, body morphology, physical activity, gut microbiota, inflammation, etc.), external exposures (radiation, infections, chemical contaminants and pollutants, diet, lifestyle factors, occupational hazards) and lastly general external exposures (social and psychological influences on the individual which also include natural build environment) [1]. Figure 1 illustrates exposomic framework that include external exposure such as lifestyle, environment and social that will influence cancer risk through changes in the multi-omics markers indicated in the diagram. Although it looked very complex but identifying risk factors by combining whole exposomic framework is beneficial for policymakers and stakeholder to formulate best cancer prevention strategies for public health.

In a pioneering study involving the sequencing of the entire tumor genome, researchers focused on a small cell lung cancer (SCLC) cell line, comparing it with genomic DNA obtained from an Epstein-Barr virus-transformed lymphoblastoid line from the same patient. The analysis unveiled more than 20,000 somatic single nucleotide variants (point mutations) [2]. Strong association between SCLC and smoking, along with the prevalence of G to T transversions as induced by oxidative stress via formation of 8-hydroxy-2-deoxyguanosine (8-OHdG), and other mutations typically associated with tobacco exposure, supported the hypothesis that this mutation spectrum represented the signature of tobacco influence across the cancer’s entire genome. In a separate report by Pleasance et al. [2010] [3], a similar approach was used to investigate mutations in a melanoma cell line. The mutational pattern in this case indicated a significant influence of sunlight, aligning with earlier data on TP53 mutation spectra.

An illustrative instance is evident in head and neck cancers, which exhibit strong connections to prior tobacco and alcohol exposure. Additionally, particularly in high-income countries, these cancers are associated with mucosal human papilloma viruses (HPV) infection. Stransky et al. conducted whole-exome sequencing on 74 tumors, revealing that HPV-positive tumors had approximately half the mutation rate compared to HPV-negative tumors, although with an overall 40-fold variation. G to T transversion mutations were prevalent, and tumors with a higher fraction of G to T mutations had more mutations in total, suggesting a general effect of tobacco mutagens [4].

Using a similar exome-sequencing approach, Agrawal et al. studied 32 tumors and found an average of 19 mutations per tumor, with a range of 2–78 [5]. Again, there were fewer mutations in HPV-associated tumors and, on average, about twice as many in tumors from tobacco users. However, unlike Stransky et al.‘s study [4], there was no enrichment for G to T transversions in this series. This could be attributed to the inclusion of a significant proportion of laryngeal tumors in Stransky et al.‘s larger study, which are less likely to be linked to HPV infection and thus exhibit higher mutation rates and G to T transversion frequencies than tumors from other sites.

Besides whole genome sequencing, exposomic study can also be identified using other biomarkers such as metabolites, gene expression changes, metagenomics and other omics approach. Combining those multi-omics approach will provide a more precise outcome in terms of providing algorithm for prediction or prognosis of disease. EPIC (European Prospective Investigation into Cancer and Nutrition) is one of the best cohorts that employs similar method and yield best outcome to understand etiology of cancer in Europe [6–7].

The application of metabolic fingerprinting analytical techniques allows us to examine the distinctive set of metabolites present in biological samples that involved in various pathophysiological processes of a disease, with a focus on cancer in the present context. However, very few studies related to metabolomics and cancer risk was conducted in Malaysia. One recently published study revealed that the metabolite profile associated with colorectal cancer (CRC) cases in Malaysia were hypoxanthine, acetylcarnitine, xanthine, uric acid, tyrosine, methionine, lysoPC, lysoPE, citric acid, 5-oxoproline, and pipercolic acid. The data also showed that the most perturbed pathways in CRC were purine, catecholamine, and amino acid metabolisms [8].

The application of exposome framework can be seen now not only in cancer studies [9–11] but also in other non-communicable diseases such as cardiovascular disease [12–14] and respiratory disease [15–17]. A study by Juarez et al. [18], used an exposome database containing more than 2000 environmental exposures from natural, built and social environment domains and they observed that exposure to ethylene oxide and ethyl dichloride, particulate matter (PM)_2.5_ and cigarette closely associated with lung cancer. Another study focusing on skin cancer suggested that physico-chemical substances, living organisms (viruses) and lifestyle factors made up exposome for this cancer [19]. A study on pancreatic cancer suggested that several exposome factors were related to pancreatic cancer alone and in combination with other exposures [20]. A recent study by Chen et al. reported that lifestyle, social and ecosystem domains are related to CRC events and incidence [21].

The study involving exposomes normally will involve big data analysis from a cohort study and multiple biomarkers. Lack of cohort study conducted in Malaysia may be one of the limitations in obtaining published evidence related to exposomes and cancer risk. Nevertheless, this field of research is still emerging and very limited epidemiological evidence reported in Malaysian setting especially focusing on cancer risk. To date, no study on whole exposomes and cancer risk has been conducted in Malaysia. Most of the studies reported are conducted individually according to the risk factors and not combining the whole exposome approach. This review will summarize studies published in this area and will identify research gap for other researchers to take opportunity and contribute to the body of knowledge.

## Main text

### Environmental exposure and cancer risk in Malaysia

The study about environmental contaminants in Malaysia is normally being conducted not in a cohort setting. Most researchers would conduct a cross-sectional study design and experimental design study where they would study contaminants in the identified source. However, there are few studies conducted on air pollution studied the impact of particulate matter with lung cancer risk.

A study by Othman et al. [22] in school classroom in Kuala Lumpur suggested that the major source of indoor dust was road dust (69%), while soil dominated the outdoor dust (74%). Health risk assessment conducted showed that the hazard quotient (HQ) value for non-carcinogenic trace metals was < 1 while the total cancer risk (CR) value for carcinogenic elements was below the acceptable limit (1.0E-06–1.0E-04) for both indoor and outdoor dust through dermal and inhalation pathways, but not the ingestion pathway. This study suggests indoor contributions of PM_2.5_ concentrations are due to the activities of the school children while the compositions of indoor and outdoor dust are greatly influenced by the soil/earth source plus industrial and traffic contribution. The major ion and trace metal concentrations in indoor and outdoor dust were Al, Fe, Zn, V, Cu and Ca^2+^ while for outdoor dominant elements and ions were Al, Fe, Zn, SO_4_^2−^, Ca^2+^ and V. Most of the studies in Malaysia conducted is via this way which is not from epidemiological evidence. However, it is worth to note that the study conducted is still important and provide preliminary data on exposure assessment.

Another study conducted from the same group in office environment showed the higher total CR value for outdoors (2.67E-03) was observed compared to indoors (4.95E-04) under chronic exposure, thus suggesting a carcinogenic PM_2.5_ risk for both the indoor and outdoor environments [23]. It was noted that the CR value reported in this study was estimated based on the inhalation unit risk (IUR), where the CR was calculated by multiplying the exposure concentration by the IUR (standard value of unit risk of 0.008 per µg m^− 3^).

A study by Sopian et al. (2021) [24] investigated the association of exposure to particle-bound (PM_2.5_) polycyclic aromatic hydrocarbons (PAHs) with potential genotoxicity and cancer risk among children living near the petrochemical industry and comparative populations in Malaysia. They collected PM_2.5_ samples using a low-volume sampler for 24 h at three primary schools located within 5 km of the industrial area and three comparative schools more than 20 km away from any industrial activity. A total of 205 children were randomly selected to assess the DNA damage in buccal cells. This study revealed that the inhalation risk for the exposed children and comparative populations was 2.22 × 10^− 6^ and 2.95 × 10^− 7^, respectively, based on the 95th percentiles of the incremental lifetime cancer risk estimated using Monte Carlo simulation [24]. The degree of DNA injury was substantially more severe among the exposed children relative to the comparative community, hence indicating that higher exposure to PAHs increases the risk of genotoxic effects and cancer among children.

Another study conducted in Rawang, Malaysia showed that the highest potentially toxic element concentration was Pb (593.3 mg/kg), whereas the lowest was Co (5.6 mg/kg) [25]. Potentially toxic elements (Cu, Cd, Pb, Zn, Ni and Cr) were linked with anthropogenic sources (urbanization process, industrial and commercial growth, urban traffic congestion). Non-carcinogenic risk by hazard index (HI) value more than 1.0 was indicated for adults and children. Similarly, carcinogenic risk by total lifetime cancer risk value also showed carcinogenic risks among adults and children.

In another study recently published [26], indoor dust is an important medium to evaluate human exposure to emerging organic contaminants. The dominant chemical groups of organic micropollutants (OMPs) of indoor dust were ascribed to n-alkanes (median: 274 µg/g), plasticizers (151 µg/g), sterols (120 µg/g), and pesticides (42.6 µg/g). The cancer risks of all OMPs were greater than 10^− 4^. This study offers a benchmark to show exposure assessment of chemicals in Malaysia.

A study was also conducted to investigate heavy metal contamination in paddy plants as this is our staple food in our country both in northern and eastern region. The health risk assessment was performed based on United States Environmental Protection Agency (USEPA) guidelines. The enrichment factor for heavy metals in the studied areas was in the descending order of Cu > As > Cr > Cd > Pb. Meanwhile, Cr and Pb exhibited higher translocation values from stem to grain compared with the others. The combined HI resulting from five heavy metals exceeded the acceptable limit (HI > 1). The lifetime CR, in both adult and children, was beyond the acceptable limit (10^− 4^) and mainly resulted from exposure. The total CR due to simultaneous exposures to multiple carcinogenic elements also exceeded 10^− 4^. It was concluded that the intake of heavy metal through rice ingestion is likely to cause both non-carcinogenic and carcinogenic health risks [27–28].

Another study conducted in 2019 [29] identified risk factors associated with lung cancer diagnosis and death by using epidemiological evidence. Lung adenocarcinoma was diagnosed in predominantly younger, female non-smokers compared to the other types of lung cancers. Lung adenocarcinoma subjects had annual PM_2.5_ that was almost twice higher than squamous cell carcinoma, small cell carcinoma and other histological subtypes. The high-risk cluster was characterized by occupational exposure to air pollution for over four hours daily, reliance on motorcycles and trucks for transportation, and a mean annual PM_2.5_ concentration. Individuals in the high-risk cluster exhibited more than five times higher risk for being diagnosed with lung adenocarcinoma (OR = 5.69, 95% CI = 3.14–7.21, *p* < 0.001) and a hazard ratio (HR) of 3.89 (95% CI = 2.12–4.56, *p* = 0.02) for lung adenocarcinoma mortality at one year. This is the only study that associated environmental exposure with risk of cancer in Malaysia based on epidemiological evidence.

### Dietary carcinogens and cancer risk in Malaysia

Publications from Malaysia were reviewed regarding various food components such as *N*-nitroso compounds, PAHs, and heterocyclic aromatic amines (HAAs), that have been identified as human carcinogens by the International Agency for Research on Cancer (IARC). Some other possible carcinogenic agents are also considered in this review, such as acrylamide and salted or other preserved foods which are common in Malaysian diet.

Carcinogenic nitrosamines, specifically *N*-nitrosodimethylamine (NDMA), *N*-nitrosodiethylamine, *N*-nitrosodibutylamine, *N*-nitrosopyrrolidine, and *N*-nitrospiperidine, are formed during the curing of meats with nitrite [30]. These nitrosamines undergo metabolic activation in the gastrointestinal tract by cytochrome P450 2E1. NDMA, in particular, generates a reactive intermediate that leads to the formation of DNA adducts, such as *N*7-methyl-2′-deoxyguanosine (*N*7-MedG), resulting in abasic site formation, DNA strand breaks, and cytotoxicity. Another DNA adduct is formed through *O*^6^-methylation of deoxyguanosine (dG), leading to *O*^6^-methyl-2′-deoxyguanosine (*O*^6^-MedG). Additionally, an endogenously formed nitrosated glycine from the consumption of red meat may induce specific mutations, such as G-A transitions and G-T transversions, in genes that promote cancer, including H-Ras and K-Ras oncogenes, as well as the p53 tumor suppressor gene [31].

Despite the increasing consumption of red meat as part of the nutrition transition in Asia, there is a notable lack of research in this area, creating a substantial research gap. No direct study has linked *N*-nitroso compound with cancer risk in Malaysia based on epidemiological evidence.

Next to be discussed is PAHs, which can originate from different sources, such as air PM and the combustion of organic material found in food. Procarcinogens like PAHs do not directly cause genotoxicity; instead, they need to undergo metabolic transformations into more reactive metabolites through xenobiotic-metabolizing enzymes, such as cytochrome P450 (CYP) 1A1/1B1, epoxide hydrolase, and aldo-keto reductase. These transformations lead to the formation of phenols, catechols, quinones, diol-epoxides, o-quinones, and radical cations [32], which can react with DNA and form DNA adducts. The risk of carcinogenesis is determined by both the level of DNA damage and the capacity to repair the damage. Carcinogenic PAHs can induce DNA damage in various human cell lines, triggering downstream DNA damage response gene products that regulate and maintain genomic stability [33]. Research exploring the association between PAHs exposure and cancer risk has been extensively conducted in China. These studies assessed the proportion of dietary intake relative to total PAHs intake and examined the correlation between dietary PAHs intake and the occurrence of abnormal lung cancer cases [34–36]. No other studies has been reported in Malaysia even related to food groups that may be associated with PAHs and cancer risk.

HAAs, which include 2-amino-1-methyl-6-phenylimidazo[4,5-*b*]pyridine (PhIP), 2-amino-3,8-dimethylimidazo[4,5-*f*]quinoxaline (MeIQx), and 2-amino-9*H*-pyrido[2,3-*b*]indole (AαC), are highly prevalent in well-done cooked meats [37]. These compounds are activation-dependent and are formed through heat-induced reactions primarily found in foods containing nitrogenous and creatine components. The quantity of HAAs is influenced by cooking temperature and the intensity of heat applied during the cooking process. HAAs can cause single strand breaks in DNA, chromosomal aberrations, and DNA adducts in guanine-rich regions. Activated metabolites can attach to DNA at the *N*^2^-position of guanine (more common) or the C8-atom of guanine (less frequent) [38]. White meat generally contains lower levels of HAAs, likely due to reduced cooking time. Another factor that may influence HAAs in white meat is the use of marinades, as different types of marinades used with chicken have been shown to impact HAAs production [39].

In terms of mechanism, HAAs undergo metabolism by cytochrome P450 enzymes, resulting in the formation of genotoxic *N*-hydroxylated metabolites. These metabolites then undergo further metabolism with conjugation enzymes, such as *N*-acetyltransferases or sulfotransferases, leading to the generation of reactive intermediates that bind to DNA, causing genomic instability that may be one of the key factors of carcinogenesis. In Malaysia, fried and grilled chicken was found to be the major dietary source of HAAs [40].

For acrylamide, the genotoxic properties of acrylamide suggest that it could potentially contribute to neoplastic transformation. The carcinogenic potential of acrylamide was demonstrated in the studies conducted by Friedman et al. in 1995. These studies revealed a notable rise in the occurrence of thyroid follicular cell adenomas and adenocarcinomas in male rats, as well as a significant increase in mammary gland fibroadenoma and adenocarcinomas in female rats [41].

In one of the studies, biomarkers of acrylamide and dietary estimates were examined, and it was observed that the consumption of crackers and chocolate showed a significant association with the concentrations of a major metabolite of acrylamide, namely *N*-acetyl-*S*-(2-carbamoylethyl)-cysteine (AAMA), in urine [42]. A large cohort study in Japan is the only study investigated on dietary acrylamide and risk in breast, gastric, lung, pancreatic, liver, colorectal, endometrial, and ovarian cancer. However, no significant association was found between dietary acrylamide and the risk of any of these types of cancer [43–47]. Perhaps Malaysian researcher should learn from the Japanese cohort to link their public cohort with dietary carcinogen. Such valuable data is really critical for public health strategies and policy making for years to come.

Notably, Hogervorst et al. [48] made an interesting discovery, demonstrating a significant correlation between dietary acrylamide intake and specific single-nucleotide polymorphisms (SNPs) in acrylamide-metabolizing enzymes, such as cytochrome P450 2E1, concerning the risk of endometrial cancer. Furthermore, they found a notable interaction between SNPs in the HSD3B1/B2 gene cluster, which affects progesterone or androgens, related to the risk of ovarian cancer.

Dietary acrylamide may also raise the risk of cutaneous malignant melanoma in men (HR: 1.13, 95% CI: 1.01–1.26) per 10 µg increment, and risk of lymphatic malignancies, such as multiple myeloma and follicular lymphoma, in men [49–50]. A very recent study published in Japan showed that dietary acrylamide has an association with risk of breast cancer through its effect on haemoglobin adducts [51]. It is known that acrylamide causes cancer owing to its mutagenic and genotoxic metabolite, glycidamide, and its effects on sex hormones. Both acrylamide and glycidamide can interact with hemoglobin to hemoglobin adducts, which may be one of the key features of cancer hallmarks.

In Malaysia, the only risk assessment study of dietary acrylamide reported is by Sharif et al. 2018 that was conducted in university students [52]. The students’ intake of coated fried chicken can give a significant health risk compared to other tested food, and all of the tested food sold in the canteen give a high number of probabilities of increased risk of cancer.

Besides known dietary carcinogen, it is worth to note on food groups and cancer risk. Limited data representing Malaysia in this field of research making this review very short. In one of the recently published case-control study, pro-inflammatory diets which contains foods that may potentially promote inflammation within the body, such as food high in sugar, refined flour, saturated fats, and red and processed meats, were reported to be associated with an increased incidence of colorectal cancer in the Malaysian population, particularly in obese subjects [53]. From the same cohort of subjects, four main dietary patterns were identified: the allergenic diet, plant-based diet, processed diet, and energy-dense diet pattern. After adjusting for potential covariates, the processed diet pattern was consistently associated with CRC (OR = 3.45; 95% CI = 1.25–9.52; *P* = 0.017) while the plant-based diet, energy-dense diet, and allergenic diet were not associated with CRC risk [54]. Another study to associate dietary pattern with colorectal adenoma risk showed that red meat consumption showed a positive association [55].

Another important evidence to note based on Malaysian landscape is related to nasopharyngeal carcinoma (NPC). Since the 1980-s, evidence on consumption of salted food with NPC were reported [56–59]. It was postulated that salt can help in the nitrosation process and may produce dangerous oncometabolites, as well as being involved in promoting Epstein Barr virus, which is a common feature in those with NPC. In an in vitro toxicity model, salt and nitroso compounds were identified as inducers of mutagenicity and genome damage [60–61]. The cancer incidence also may be related to the genetic polymorphism, Epstein-Barr virus and many other confounding factors that should be included in the risk factors by using the exposome model framework which warrants further investigation in this area of research.

## Top of form

### Occupational hazards and cancer risk in Malaysia

Malaysia’s diverse economy encompasses various industries, some of which expose workers to potential carcinogens such as asbestos, solvents, and heavy metals. Workers in these industries may face elevated cancer risks related to their specific occupational exposures.

To date, there is no direct study reported on occupational hazard and exposure with cancer risk or any cancer hallmarks. However, there is one study related to exposure of radiation in tin by-product processing industry workers reported in Malaysia [62]. With poorly established safety and health practices in operating plant, amang poses extremely high radioactivity problem associated with high occupational ionizing radiation exposures to workers. The study found out that for 8 h of work time, a worker is estimated to receive an average effective dose of 0.1 mSv per day from external γ radiation source with a maximum up to 2 mSv per day for extreme exposure situation.

Interferences of different exposure routes for examples inhalation of equivalent equilibrium concentration (ECC) of ^222^Rn and ^220^Rn progenies and airborne long-lived α particles from the dusty working environment could pose a higher total effective dose as much as 5 mSv per day and 115 mSv per year. The value is 5 times higher than the annual dose limit for designated radiation worker (20 mSv) in Peninsular Malaysia. The study found that 41% of the total received an effective dose received by a worker was contributed by ^222^Rn, 32% of airborne particulates and dust, 23% from external γ exposure and 4% from ^220^Rn. This rare earth element is a known genotoxic agent and may contribute to one of the key components in cancer hallmarks which is genomic stability.

Further findings from Malaysia investigated genetic damage due to occupational exposure in auto repair workshop workers. Micronucleus frequency, comet tail length and relative telomere length differences were significantly greater in the auto repair workshop workers. Duration of working time was significantly associated with micronucleus frequency, comet tail length and relative telomere length [63]. Another study investigating workers and genotoxic effects revealed that paddy farmers who chronically exposure to a mixture of organophosphates has at least 2-fold significant increase of DNA damage as compared with control group [64].

Lack of data in this focus area warrants further investigation as occupational hazard and exposure are one of the main exposures for cancer carcinogenesis.

### Infectious agents and cancer risk in Malaysia

Certain infections, such as *Helicobacter pylori* (linked to stomach cancer) and the HPV (linked to cervical and other cancers) infections, represent significant cancer risk factors in Malaysia.

Based on published evidence, it has been shown that *Helicobacter pylori* may be associated with the increased risk of gastric cancer, particularly in relation to high salted food consumption by Malaysian population [65].

In Malaysia, cervical cancer is the third leading cause of female cancer deaths and the second most common cancer that occurs in women aged 15 to 44 years old with 621 deaths annually [66]. HPV are small non-enveloped viruses, with a double-stranded circular DNA genome. This virus is oncogenic and caused persistent infection in the human system, hence leading to carcinogenesis. The two most important proteins in the carcinogenesis of cervical cancer are E6 and E7, which are responsible for the dysregulation of the cell cycle [67]. A study by Rahmat et al. 2021 showed that the most common high-risk HPV type among women living in urban areas in Malaysia is HPV 52, which is not the type of infection the current HPV vaccine is covered for protection among females [68]. This data is very important because it might evidence to the stakeholders on why cervical cancers is still increasing in Malaysia despite the vaccines given.

### Socioeconomic factors and cancer risk in Malaysia

Disparities in socioeconomic status and access to healthcare can influence cancer risk and outcomes. Low socioeconomic status may lead to increased exposure to environmental carcinogens and limited access to early detection and treatment services. Despite looking thoroughly on socioeconomic factors and cancer risk in Malaysia, only one study reported on HPV infection which reflects cervical cancer that was significantly associated with employment (OR 4.94; CI 1.58–15.40) and education at secondary/high school level [69]. Only few studies have focused on the influence of social determinants on the risk of cancer. Hastert et al. used data from the Vitamins and Lifestyle Study to examine the relationship between socioeconomic status and CRC incidence. Living in the lowest socioeconomic status areas was associated with a higher CRC incidence than those living in the higher socioeconomic status areas [70]. This can be due to the access of medical facility.

A comprehensive review by Carethers and Doubeni in 2020 showed that a disadvantaged socioeconomic position is related to an increased risk of CRC [71]. Social support was another social factor that affected colorectal cancer carcinogenesis where lower social support was associated with a higher incidence of CRC [71]. Additionally, social support reduces stress and depression. Social support raises the esteem of individuals and makes them feel valued; therefore, they may take better care of themselves and be more receptive to preventative services. They also may experience lesser stress hormones to reduce the risk of immune dysregulation, thereby suppressing the environment for tumour initiation.

It is obvious that in Malaysia, social and economic factors should be included in any of the exposure assessment related to health impact in this case carcinogenesis. Perhaps it is due time that we relook again at how we design our study to incorporate all of the exposomes.

## Conclusion

**Fig. 2:**
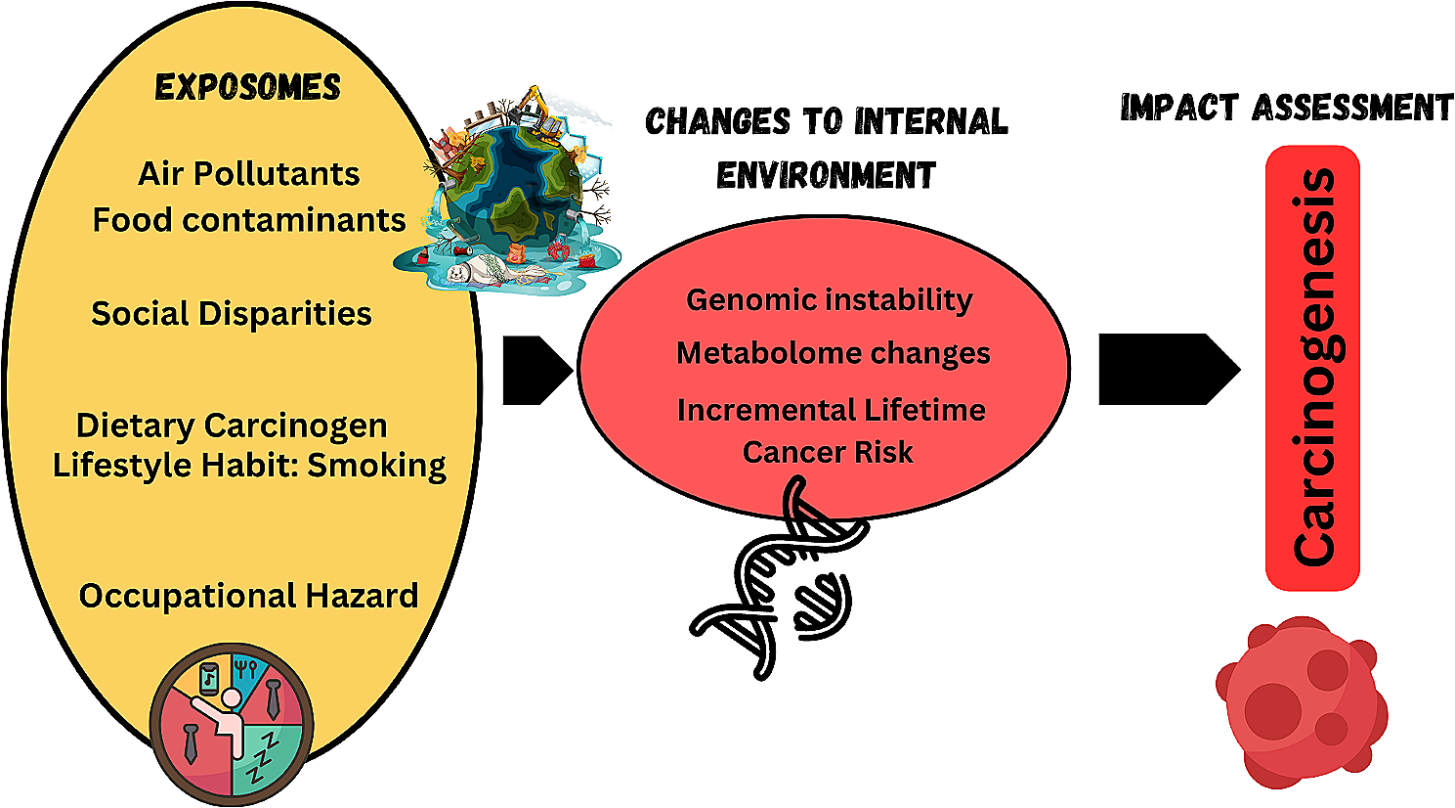
Mapping exposomes and cancer risk in Malaysian landscape

Designing exposomic framework to understand cancer carcinogenesis is really critical and important to understand. As listed in this review, there is a huge gap in epidemiological evidence in relation to any omics marker and cancer risk integrated with any of the exposomes, especially in Malaysia. Figure 2 illustrates exposomic map of Malaysian data based on cancer risk published. This creates research gap for Malaysian researchers and promote global collaborations with other researchers.

This review is of course with a lot of limitation due to limited data published in Malaysia. Perhaps this article will assist to shed lights to cancer and toxicology researchers who wants to understand the complex interplay between environmental-lifestyle and carcinogenesis. Nevertheless, understanding the exposome and its relation to cancer risk in Malaysia is of paramount importance for several reasons:

1. Unique Exposures: Malaysia’s population is exposed to a distinctive set of environmental factors, lifestyle choices, and dietary habits that may differ from other regions. Investigating the specific exposome in Malaysia can uncover novel carcinogens and risk factors that are relevant to the local population.
2. High Cancer Burden: Cancer is a significant public health concern in Malaysia, with a substantial number of cancer cases diagnosed each year and present huge socioeconomic burden for Malaysian government. Identifying the key environmental and lifestyle factors contributing to cancer risk can help implement targeted preventive measures and interventions to reduce the cancer burden.
3. Prevention Strategies: By comprehensively studying the exposome and its link to cancer risk, policymakers and healthcare professionals can develop effective preventive strategies tailored to the Malaysian population. This may include awareness campaigns, lifestyle modifications, and environmental regulations to minimize cancer risk factors.
4. Precision Medicine: Understanding the exposome’s impact on cancer risk can enable personalized approaches to cancer prevention and treatment. By considering individual variations in exposure, genetics, and lifestyle, healthcare providers can offer more targeted and effective interventions.
5. Environmental Sustainability: Research on the exposome can also shed light on environmental hazards and pollution that may contribute to cancer risk. Addressing these issues can promote environmental sustainability and protect both human health and the ecosystem. It is critical time for all the stakeholders and ministries to work together for this purpose.
6. Global Research Collaboration: Studying the exposome and cancer risk in Malaysia contributes to the broader global effort to understand cancer etiology. Collaborative research can lead to shared knowledge and strategies for cancer prevention and control worldwide.

Overall, investigating the exposome and its relation to cancer risk in Malaysia is essential for improving public health, reducing cancer incidence, and advancing cancer research and prevention strategies in the region and beyond.

## Data Availability

Not applicable.
